# Informal Volunteering Helps Older Adults Stay Put Too! But, Does Gender Matter?

**DOI:** 10.1093/geroni/igaa057.164

**Published:** 2020-12-16

**Authors:** Huei-wern Shen, Tam Perry

**Affiliations:** 1 National Taiwan Normal University, Sintien, New Taipei, Taiwan (Republic of China); 2 Wayne State University, Detroit, Michigan, United States

## Abstract

Many older adults desire to remain in one’s home for as long as possible, and many factors have been identified to be helpful, such as formal volunteering (doing unpaid work for religious, educational, health-related or other charitable organizations). While many older adults volunteer formally, many others volunteer informally (providing unpaid help to friends, neighbors, or relatives who did not co-reside). However, less is known about the relationship between informal volunteering and relocation. Guided by the social and material convoy framework, the present study explores the intersection of gender, informal volunteering, and relocation (no move, move within area, and move out of area). Utilizing data from 2008 and 2010 Health and Retirement Study, 8,361 older adults who were 65 and above in 2008 were included. When older people’s financial resources, health, environment, and demographics were controlled, findings from multinomial logistic regression showed that older adults who volunteered informally were less likely to move within area two years later. When stratified by gender, it was found that female (n=4,832) volunteered informally in 2008 were less likely to move within area within two years, too; whereas for male (n=3,529), those who informal volunteered in 2008 were less likely to move out of area in 2010. According to the findings, informal volunteering helps older adults stay put. Future research is needed to understand why informal volunteering helps reduce short distance moves for women but helps reduce long distance moves for men.

